# Relationship between mechanical dyssynchrony and intra-operative electrical delay times in patients undergoing cardiac resynchronization therapy

**DOI:** 10.1186/1532-429X-16-4

**Published:** 2014-01-06

**Authors:** Jonathan D Suever, Gregory R Hartlage, R Patrick Magrath III, Shahriar Iravanian, Michael S Lloyd, John N Oshinski

**Affiliations:** 1 Wallace H. Coulter Department of Biomedical Engineering, Georgia Institute of Technology / Emory University, 1364 Clifton Road, Suite AG30, Atlanta, GA 30322, USA; 2 Division of Cardiology, Department of Medicine, Emory University School of Medicine, Atlanta, GA, USA; 3 Department of Radiology & Imaging Sciences, Emory University School of Medicine, Atlanta, GA, USA

**Keywords:** Electrophysiology, Cardiac resynchronization therapy, Cardiovascular magnetic resonance, Electromechanical delay

## Abstract

**Background:**

It is important to understand the relationship between electrical and mechanical ventricular activation in CRT patients. By measuring local electrical activation at multiple locations within the coronary veins and myocardial contraction at the same locations in the left ventricle, we determined the relationship between electrical and mechanical activation at potential left ventricular pacing locations.

**Methods:**

In this study, mechanical contraction times were computed using high temporal resolution cine cardiovascular magnetic resonance (CMR) data, while electrical activation times were derived from intra-procedural local electrograms.

**Results:**

In our cohort, there was a strong correlation between electrical and mechanical delay times within each patient (R^2^ = 0.78 ± 0.23). Additionally, the latest electrically activated location corresponded with the latest mechanically contracting location in 91% of patients.

**Conclusions:**

This study provides initial evidence that our method of obtaining non-invasive mechanical activation patterns accurately reflects the underlying electromechanical substrate of intraventricular dyssynchrony.

## Background

Intraventricular dyssynchrony results from the dyscoordinate contraction of the ventricular walls, and the presence of dyssynchrony is associated with increased mortality in heart failure patients [1]. Cardiac resynchronization therapy (CRT) is a treatment option for drug-refractory heart failure patients with low ejection fraction and evidence of left ventricular (LV) electrical dyssynchrony. Currently, the primary marker for LV electrical dyssynchrony is QRS duration. A QRS duration of 120 ms or greater is indicative of delayed electrical activation within the ventricles and is an independent predictor of mortality and sudden death in heart failure patients [2]. However, at least 30% of patients fail to improve after CRT device implantation despite appropriate patient selection [3]. One reason for this non-response may be attributed to a difference between electrical and mechanical dyssynchrony measurements. QRS duration is a measure of *electrical* dyssynchrony and may not accurately reflect the degree of *mechanical* dyssynchrony seen in imaging techniques such as CMR or echocardiography [4]. Currently, it is not clear if the presence of electrical and mechanical dyssynchrony is equivalent in determining patient response to CRT.

There are two reasons that the relationship of electrical and mechanical dyssynchrony is important in CRT. First, the effect of left ventricular (LV) lead position has been studied extensively in an attempt to explain poor response to CRT. Several groups have shown that pacing the LV in the latest contracting segment (greatest *mechanical* dyssynchrony) results in improved response to CRT [5-9]. However, other groups have demonstrated that pacing in the most delayed region does not result in better response [10,11]. Similarly, canine and human studies have seen varied results when pacing at the latest electrically activated region [12,13]. These contradictory findings may be a result of the complex interaction between electrical and mechanical dyssynchrony.

Second, many groups have shown that the time to mechanical contraction in a region is longer than the underlying electrical activation time [14,15]. This delay is often termed the electromechanical delay (EMD), and it is the delay between electrical activation and onset of shortening (contraction). EMD has been shown to vary regionally within the LV as well as vary between individuals [16]. Therefore, any relationship between electrical and mechanical activation times may not be applicable across a range of patients. EMD is often accentuated in late-contracting regions due to the larger dP/dt within the LV caused by the early-contracting segments. The larger EMD is a result of the increased force required to generate shortening within the late activated region [15]. Interestingly, there can even be a mechanical delay without any evidence of electrical activation delay [17]. Most of these studies, however, have been conducted in canine models of LBBB and it is uncertain how this relationship manifests in patients undergoing CRT.

The purpose of this study was to combine regional mechanical contraction timing information throughout the LV derived from cardiovascular magnetic resonance (CMR) with measures of electrical activation delay derived from local electrograms acquired at several locations in the LV from within the coronary veins. After registering electrogram measurements to the CMR-derived maps of regional dyssynchrony, we compared electrical activation times and mechanical contraction times at potential LV pacing locations to evaluate how electrical and mechanical timing varies by location in the LV and between individuals.

## Methods

This study was conducted in eleven patients enrolled for CRT based upon current guidelines (QRS Duration > 120 ms, Ejection Fraction < 35%, and NYHA Heart Failure Class III-IV despite optimal medical therapy). Twelve-lead EKGs were used to classify all patients based upon their QRS morphology. This study was approved by Emory University's Institutional Review Board (IRB) and all patients gave written informed consent.

### CMR acquisition

Patients were placed in a 1.5 T Siemens Avanto (Siemens Healthcare, Erlangen, Germany) scanner and were imaged using a 5-element phased array coil. EKG triggering was used to obtain 60 frames per cardiac cycle yielding a temporal resolution of 13.7 ± 3.3 ms (Range: 8.9 – 19.6 ms). Steady-state free precession (SSFP) short-axis images were acquired parallel to the mitral valve plane to cover the entire length of the LV at a slice thickness of 7 mm and no slice gap. Two- and three-chamber cine images were also acquired. Acquisition parameters: acquisition matrix size = 192x192, reconstructed matrix size = 192x192, field of view = 275x275 mm, flip angle = 67º, TR = 4 ms, TE = 1.3 ms. In addition to SSFP cine images, a short-axis stack of late gadolinium enhancement (LGE) images were acquired at the same locations as the cine images to identify the presence of myocardial scar.

### Regional mechanical delay maps

**Figure 1:**
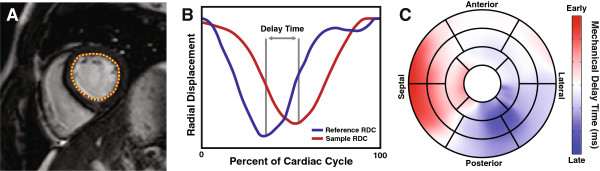
**Mechanical Delay Times from Magnetic Resonance Imaging.** Endocardial contours are traced on short-axis cine SSFP images **(A)** and the distance relative to the centroid is computed **(B; red)**. Each regional RDC is compared to a patient-specific reference (B; blue) to determine the mechanical delay time. These delay times are then mapped to the standard AHA 17 segment model **(C)**.

To determine the mechanical delay times throughout the LV, a previously-developed method was employed [18]. Briefly, endocardial boundaries were semi-automatically traced on all of the short-axis images (Figure 1A). Radial displacement curves (RDCs) were generated by computing the distance of the endocardial contour relative to the centroid of the LV at 100 circumferentially spaced points (Figure 1B). To account for translation of the LV over the cardiac cycle, the centroid was determined from the location of the mitral valve annulus and apex of the LV in the two and three-chamber views. RDCs were sorted into similarly-contracting groups using QT clustering [19] and a patient-specific reference RDC was obtained by averaging all members of the largest cluster. Normalized cross-correlation [20] was then used to determine the temporal delay between each RDC throughout the LV and the reference RDC (Figure 1B). By using the cross-correlation of the curves, the resulting mechanical delay time reflects the delay over the entire cardiac cycle. After mechanical delay times had been computed throughout the entire LV, the delay times were then mapped to a polar map (bullseye) display and the standard AHA 17-segment model was superimposed [21] to facilitate comparison between electrical and mechanical delay times (Figure 1C).

### Electrogram acquisition

One of several models of approved passive fixation LV leads was used to obtain local electrograms (EGMs) during the CRT device implantation procedure. The RV pacing lead was inserted into the apex of the RV (near the apical septal wall of the LV) and the resulting RV EGM was recorded. No RV pacing was applied during EGM measurement. After coronary sinus venography, the LV lead was directed to various achievable locations throughout the coronary venous system that were potential pacing sites. All local EGMs were recorded in a unipolar configuration. EGMs were simultaneously recorded from the RV and LV leads at a sampling frequency of 500 Hz (2 ms temporal resolution).

### Regional electrical delay

Electrical delay times were assessed as the delay between the RV and LV EGMs as determined using in-house software written in Matlab (The MathWorks, Natick, Massachusetts). Each EGM was first filtered using a band-pass filter. Low frequencies were removed to eliminate baseline drift that could occur over the course of the 5 sec measurement duration. Additionally, an upper limit was selected to eliminate high-frequency noise from the signal.

**Figure 2:**
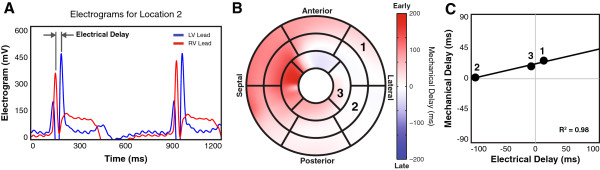
**Comparison of Electrical and Mechanical Delay Times.** Electrical delay times at each lead location were determined by comparing the LV EGM with the RV EGM **(A)**. Using the mapped lead positions, mechanical delay times could be sampled from the regional dyssynchrony map **(B)** and compared directly to the electrical delay times **(C)**.

LV EGMs were super-imposed on the RV reference EGM for each location. A blinded experienced observer selected corresponding peaks of the two signals (the arrival of electrical activation at each site). The peak-to-peak time difference was computed from these user-defined points to obtain the electrical delay time at each sampling location (Figure 2A).

This study utilized the convention that a positive electrical delay time indicated that the lateral wall was activated prior to the septal wall, whereas a negative electrical delay time indicated that the activation was delayed in the lateral wall relative to the septal wall.

### Coronary venous imaging

**Figure 3:**
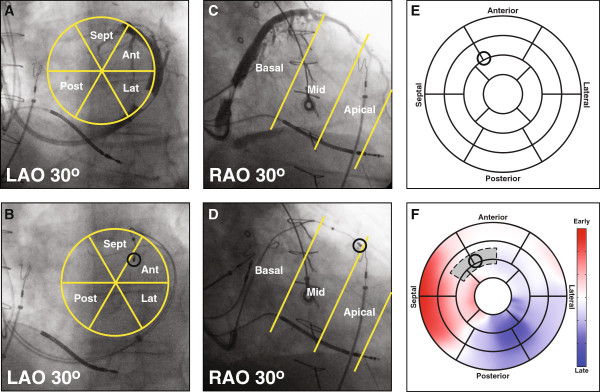
**Left Ventricular Lead Localization.** Biplane venograms **(A, C)** and lead localizing biplanes **(B, D)** were used to map LV pacing lead locations (black circle) onto the AHA 17-segment model **(E)**. LAO images were used to determine the circumferential location while RAO images were used for longitudinal position. Mechanical delay times in a region surrounding the lead location (shaded region) can be determined directly from the 17-segment model **(F)**.

Venous imaging in the electrophysiology lab was performed using standard procedures. Briefly, prior to any EGM measurements, a balloon catheter was inflated to occlude the coronary sinus and iodinated contrast agent (Visipaque, GE Healthcare, Waukesha, WI) was injected retrograde into the coronary sinus. During contrast injection, coronary venograms were acquired at the following views: 30º right-anterior-oblique (RAO), anterior-posterior (AP), and 30º left-anterior-oblique (LAO) (Figure 3, top row). Each LV EGM recording site was recorded fluoroscopically in RAO and LAO 30° positions (Figure 3, bottom row).

### Registration of lead position to regional mechanical delay maps

To determine the mechanical delay corresponding to each of the locations where the EGM was measured, it was necessary to map the lead location from the angiographic images to the CMR domain. An experienced observer used biplane venograms and lead localizing biplane images to determine the location of the LV pacing lead on the AHA 17-segment model using Mortensen’s o’clock technique (Figure 3) [22]. The RAO image was used to determine the longitudinal position of the lead, while the LAO image was used to determine the circumferential position. Lead positions were determined for all coronary veinEGM recording sites.

### Corresponding electrical and mechanical delay times

Since the CMR-derived mechanical delay times are already projected onto the AHA 17-segment model, it is possible to directly compare the mechanical and electrical delay times at each location (Figure 3F). Due to the lower spatial resolution of the local EGM, and to reduce sensitivity to possible errors in lead localization, mechanical delay times within a region the size of an AHA segment, but centered about the lead location, were averaged to obtain the mechanical delay time corresponding to each lead position (Figure 2B). The mechanical and electrical delay times obtained at each location were then compared (Figure 2C).

### Comparison of electrical and mechanical delays

Pearson’s correlation coefficient was computed between the electrical and mechanical delay times observed for each patient. The location (AHA segment number) of the latest electrical activation and latest mechanical contraction were determined in each patient.

The CMR-derived regional mechanical delay times were computed relative to a patient-specific reference curve. The EGM-derived electrical delay times in the LV were computed relative to the electrogram obtained at the apex of the RV. The different references for the electrical and mechanical measurements imply that: 1) the exact numerical values of the electrical and mechanical delay measurements at a single location cannot be compared, and 2) each patient has a different reference, so mechanical (or electrical) measurements cannot be compared between patients. As a result, the correlations between electrical and mechanical delays were done on each patient individually.

## Results

Eleven patients enrolled for CRT underwent a CMR examination before the procedure and multi-site electrogram acquisition during the CRT device implantation procedure without incident. All patients presented with abnormal left ventricular conduction abnormalities, seven of which had strict left bundle branch block (LBBB). No patients had a previous myocardial infarction as determined from LGE imaging. Mechanical and electrical delay times were sampled at a total of 40 potential pacing sites (14 anterolateral, 16 posterolateral, and 10 anterior) within the coronary veins of the eleven patients. All patients had at least 3 locations measured.

Correlation values (R^2^) between electrical and mechanical delay times on a per-patient basis were 0.78 ± 0.23 (0.35 – 1.00).

**Figure 4:**
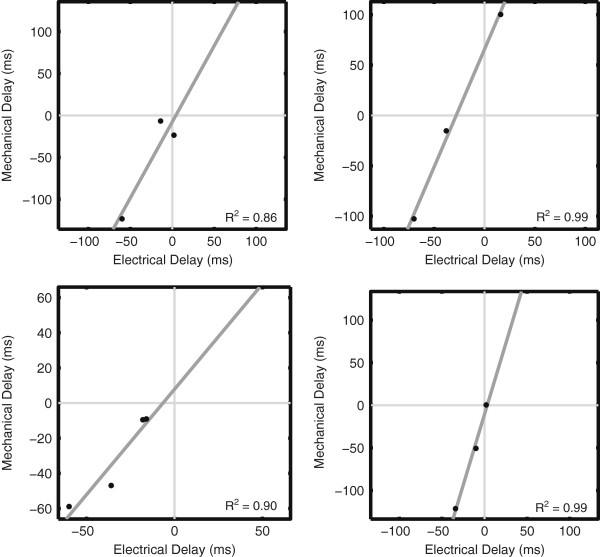
**Relationship Between Electrical and Mechanical Delay Times.** The relationship between electrical and mechanical delay times is shown for four representative patients. All trendlines exhibited high and positive correlation values.

There was a strong linear relationship between electrical and mechanical delay times in all patients. The positive slope of this best-fit line for all patients indicated that as electrical delay increases, the mechanical delay also increases. However the slope and hence the magnitude of this relationship varies from patient to patient (Figure 4). The slope of the trendline was 1.24 ± 1.02, indicating that mechanical delay was greater than the corresponding electrical delay. Additionally, among the LV EGM sites, the segment that had the largest electrical delay also had the largest mechanical delay in 10 of the 11 patients (91%). The latest segment was posterolateral in 5 patients, anterolateral in 5 patients, and anterior in 1 patient.

## Discussion

The main findings of this study were: 1) there was a strong correlation between electrical and mechanical delay times measured at potential pacing locations within the coronary veins; 2) the relationship between electrical and mechanical dyssynchrony varied greatly between patients, but was always positive; 3) the site with the largest electrical delay was the same site with the largest mechanical delay in 91% of patients; and 4) the latest activated and contracting site is often not the posterolateral wall.

A number of studies have measured the electromechanical delay in the LV, however, most have utilized animal or computational models for their analysis [13,15,16,23,24]. Regional analysis has shown that the temporal delay between electrical activation and the onset of shortening varies depending upon location in the LV [16]. In a computational model of the canine heart, Usyk et al. found that there was no direct correlation between electrical activation time and the onset of shortening. There were several cases where the EMD was positive, indicating that mechanical contraction actually occurred prior to electrical activation [24]. Other canine studies have also shown that mechanical activation follows electrical activation with a non-fixed delay which could result in a poor correlation between the two variables [15,16]. In models of LBBB, this variation in electromechanical delay is attributed to the requirement that the late lateral wall has to overcome the higher dP/dt values due to early septal contraction [15]. Biventricular pacing results in a decreased and more consistent EMD, but this isn’t always accompanied by acute improvement in LV ejection fraction or QRS duration [13].

Our study showed a strong correlation between electrical and mechanical delay times at different sites within the LV. We employed a method that utilized the entire radial contraction curve in an attempt to characterize LV dyssynchrony rather than just using a single time point measuring the onset of shortening to determine mechanical contraction time. Additionally, we only utilized potential pacing sites located throughout the coronary sinus. Had we considered locations within the septum, it is possible these data points wouldn’t agree with the previously-stated trend.

The slope of the fit to the plot of mechanical vs. electrical delay time provides a measure of the type of relationship between the two. Not only was there a strong linear relationship between electrical and mechanical delay times at the sampling locations, but the relationship was always positive, indicating that increasing electrical delay results in an increased mechanical delay. A larger slope indicates a longer mechanical delay for a small electrical delay whereas a slope less than one implies that even large electrical delays don’t result in substantial mechanical delays. Delay times were only compared between sites within each patient. It is important to note that although there was a consistent positive relationship between the two, the slope values were 1.2 ± 1.0(Range: 0.06 – 3.32), therefore the relationship between electrical and mechanical delay times was very dependent upon the individual. No observable trend existed when data from all patients was combined.

In our study we found that the latest electrical region corresponded with the latest mechanical region in ten of the eleven patients. In the one patient in whom this relationship did not hold true, the latest segments electrically and mechanically were neighboring posterolateral segments.

Previous studies have come to a wide range of conclusions regarding whether pacing the latest electrically or mechanically activated site improves response to CRT [5,7,9,11-13]. A variety of modalities have been used to assess both electrical and mechanical dyssynchrony in the LV. Several studies have demonstrated that pacing in the latest contracting segment results in improved CRT response [5-9] by employing electro-anatomical mapping to measure endocardial electrical activation throughout the entire LV [12,15]. Tse et al. found that pacing at the latest activated region in the posterior and lateral wall of the LV resulted in improved acute global response [12]. In Russell’s canine study, however, electrical activation patterns and QRS duration correlated poorly with global measures of LV function [13]. Using CMR techniques that have been shown to be better predictors of mortality in heart failure patients [25,26], Foley et al. found the location of the LV pacing lead to have little effect on response [11]. These discrepancies could be due to the fact that some of these studies relied upon irreproducible echocardiographic measures of mechanical dyssynchrony and assessed patient response at 6-month time points. We also feel that the mixed results from these previous studies could be accounted for by the heterogeneity amongst individuals that we observed in this study.

In our current study, only epicardial electrical activation was measured with the LV pacing lead. The current approach for LV lead placement involves epicardial placement of the lead via the coronary veins, therefore identification of the latest electrical region in the *entire* LV is not as important as finding the latest site that is accessible via the coronary sinus and its tributaries, unless surgical epicardial lead placement is being considered. Our analysis showed very good agreement between the latest electrically activated site measured by EGM and the CMR-derived mechanical data. Therefore, either electrical or mechanical delay times can be used for optimizing CRT LV lead placement. We found that by analyzing *only* potential pacing sites, there is a strong correlation between electrical and mechanical delay times. It is important to note that a number of factors, in addition to dyssynchrony, have been shown to affect an individual’s response to CRT [27-30].

In several patients, both electrical and mechanical delay times were very small indicating no dyssynchrony. This may mean that we did not acquire an EGM in an area with delayed electrical or mechanical activation. For example, in one LBBB patient who had a particularly poor correlation value (R^2^ = 0.35), there were 3 sampling sites located in posterolateral, anterolateral, and anterior segments. The mechanical delay times amongst these sites ranged from −2 to 26 ms, while the electrical delay times ranged from −2 to 6 ms. Both of these ranges are small relative to the temporal resolution of either modality, therefore resulting in a potentially inaccurate correlation between the two measurements. If you exclude this patient from analysis, the average correlation value for LBBB patients rises to 0.83.

There were two patients with very low slope values (0.06 and 0.23) but with good correlation values (0.98 and 0.83, respectively). In these patients, a large electrical delay time yielded relatively constant mechanical delay times. Using computation models, it has been reported that mechanical contraction can occur prior to epicardial breakthrough of the electrical activation front [24].

### Limitations

Instead of measuring onset of shortening, our method computes the mechanical delay by comparing the entire radial contraction curve of a segment to the patient-specific reference. This means that a patient may have a slow radial contraction of the myocardium in a particular region despite having an early onset. The advantage of this method is that we can determine the timing of the true contraction of the segment relative to the rest of the LV; however, our method may be incapable of detecting early inward motion of the septum but rather identifies the later thickening of the septal wall.

We only have an average of 3.6 measurement locations per patient, resulting in an obviously low sampling density. This would be difficult to improve due to extra time and radiation required for additional data points. Effort was made to sample a wide range of regions in each patient to compensate for this limitation.

Additionally, we only had nine subjects with strict LBBB enrolled in the study. The invasive nature of the procedure and additional fluoroscopic imaging prevent the enrollment of large numbers of participants. Despite the small sample size, the relationship between electrical and mechanical dyssynchrony was very consistent.

## Conclusion

We found that there is a strong positive correlation between electrical and mechanical delay times in the LV pacing sites accessible via the coronary veins. Additionally, the latest electrical region corresponded with the latest mechanical region in 91% of patients. This preliminary study provides initial evidence that pacing guided by non-invasive mechanical activation patterns targets the electromechanical substrate of intraventricular dyssynchrony.

## Competing interests

The authors declare that they have no competing interests.

## Authors’ contributions

JS helped with the study design, developed the software, performed processing and analysis and drafted the manuscript. GH, RPM, and SI assisted in all aspects of data acquisition, processing, and analysis. ML and JO helped to conceive of the study, participated in its design, and assisted with data acquisition. All authors assisted in editing and approving the final manuscript.
